# Nomograms for postsurgical extrahepatic recurrence prediction of hepatocellular carcinoma based on presurgical circulating tumor cell status and clinicopathological factors

**DOI:** 10.1002/cam4.6178

**Published:** 2023-06-20

**Authors:** Hao‐Wen Wei, Shui‐Ling Qin, Jing‐Xuan Xu, Yi‐Yue Huang, Yuan‐Yuan Chen, Liang Ma, Lu‐Nan Qi

**Affiliations:** ^1^ Department of Hepatobiliary Surgery Guangxi Medical University Cancer Hospital Nanning China; ^2^ Key Laboratory of Early Prevention and Treatment for Regional High Frequency Tumor, Ministry of Education Nanning China; ^3^ Department of Ultrasound First Affiliated Hospital of Guangxi Medical University Nanning China; ^4^ Guangxi Liver Cancer Diagnosis and Treatment Engineering and Technology Research Center Nanning China

**Keywords:** circulating tumor cells (CTC), extrahepatic recurrence (EHR), hepatic resection, hepatocellular carcinoma (HCC), prediction model

## Abstract

**Background and Aims:**

Extrahepatic recurrence (EHR) is one of the major reasons for the poor prognosis of hepatocellular carcinoma (HCC). The present study aimed to develop and assess the performance of predictive models by using a combination of presurgical circulating tumor cell (CTCs) data and clinicopathological features to screen patients at high risk of EHR to achieve precise decision‐making.

**Patients and Methods:**

A total of 227 patients with recurrent HCC and preoperative CTC data from January 2014 to August 2019 were enrolled. All patients were randomly assigned to one of two cohorts: development or validation. Two preoperative and postoperative nomogram models for EHR prediction were developed and multi‐dimensionally validated.

**Results:**

Patients with EHR had generally lower recurrence‐free survival (*p* < 0.001), and overall survival (*p* < 0.001), and significantly higher CTC counts (epithelial CTCs, epithelial/mesenchymal hybrid CTCs, and mesenchymal CTCs count, all *p* < 0.05) than those without EHR. Univariate and multivariate analyses revealed that EHR was associated with four risk factors in the development cohort: total CTC count (*p* = 0.014), tumor size (*p* = 0.028), node number (*p* = 0.045), and microvascular invasion (*p* = 0.035). These factors were incorporated into two nomogram models (preoperative and postoperative), which reliably predicted EHR through multidimensional verification (e.g., calibration plot, receiver operating characteristic analysis, decision curve analysis, and clinical impact curve analysis) in the development and validation cohorts, respectively. With threshold of scores of 100.3 and 176.8 before and after surgery respectively, both nomograms were able to stratify patients into two distinct prognostic subgroups (all *p* < 0.05).

**Conclusion:**

The present study proposed two nomogram models integrating presurgical CTC counts and clinicopathological risks and showed relatively good predictive performance of EHR, which may be beneficial to the clinical practice of HCC recurrence. Further multicenter studies are needed to assess its general applicability.

## Graphical abstract

This study aimed to develop and assess nomogram models prediction of postsurgical extrahepatic recurrence (EHR) in patients with hepatocellular carcinoma. This study developed and assessed the performance of predictive models via presurgical circulating tumor cell data analysis and multiple clinicopathological features in patients with a high risk of developing EHR.

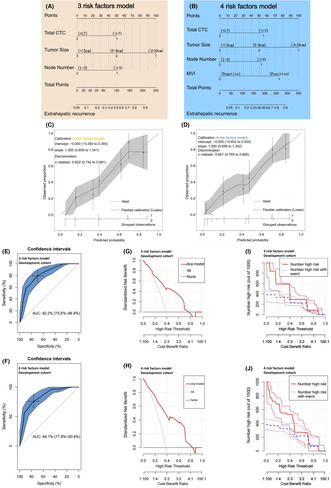

## 1 INTRODUCTION

The most prevalent type of liver cancer is hepatocellular carcinoma (HCC), which is also one of the top causes of cancer‐related death globally.^1^, ^2^, ^3^ Hepatic resection is the first‐line treatment for HCC, and advances in preoperative assessment have improved the surgical outcomes of patients undergoing hepatic resection for HCC. However, the high incidence and pattern multiplicity of postsurgical recurrence severely restrict long‐term postoperative prognosis.^4^ Therefore, accurate prediction and effective control of postsurgical recurrence to improve the overall prognosis of HCC have become an important focus of medical practice.

Postoperative recurrence patterns vary among patients. In general, approximately 80% of HCC cases recur intrahepatically, including solitary intrahepatic and multiple intrahepatic tumors.^5^ Several patients experience intrahepatic recurrence accompanied by extrahepatic recurrence (EHR).^6^, ^7^, ^8^ In addition, approximately 15% of recurrent HCCs were extrahepatic.^8^ In our previous study, based on a large‐sample multicenter analysis of HCC recurrence patterns, we identified four recurrence subtypes (Qi's) that combined recurrence features, survival, and potential therapeutics after recurrence: Type I (solitary intrahepatic oligorecurrence), Type II (multi‐intrahepatic oligorecurrence), Type IIIA‐IIIC (progression recurrence), and Type IVA‐IVB (hyper‐progression recurrence).^9^ EHR corresponds exactly to Type III or IVB recurrence, accounting for approximately 33% of all recurrences. Although EHR is not the predominant type of HCC recurrence, it nevertheless has a very dismal prognosis because there is no curative therapy available. Thus, identifying risk factors to achieve a good prediction of EHR is vital for improving long‐term survival outcomes for patients with HCCs.

When compared to HCC without EHR, postsurgical EHR is multifactorial and may exhibit poor biological behavior. Studies have indicated that the development of postsurgical EHR of HCC is influenced by multiple clinicopathological features, including large tumor size, high AFP level, incomplete tumor capsule, portal vein tumor thrombus (PVTT)/MVI status, positive for HBsAg, high blood loss during surgery, as well as some molecular events (e.g., P53 mutations and CD44v3 expression).^10^, ^11^, ^12^, ^13^, ^14^ In addition, circulating tumor cells (CTCs) derived from primary tumors are involved in the hematogenous metastatic spread of HCCs to remote locations, making them a potential source of EHR after surgery. Our previous research found that a high preoperative CTC count reflects the aggressiveness of a solid tumor and is an important predictor of multi‐intrahepatic and EHR.^7^, ^15^ However, few ideal postsurgical EHR predictive models have been generated based on the risk variables selected from these clinicopathologic factors. Therefore, in the current study, we sought to develop and assess the performance of predictive models by using a combination of presurgical CTC data analysis and multiple clinicopathological features to screen patients with a high risk of EHR to achieve precise treatment of HCC.

## 2 MATERIALS AND METHODS

### 2.1 Population under study

A total of 227 HCC patients with definitive recurrence information who had undergone a macroscopic R0 resection at the Guangxi Medical University Cancer Hospital in Nanning, Guangxi Province, China, between June 2014 and May 2019 were enrolled. The inclusion criteria were: (1) a conclusive pathological diagnosis of HCC according to the world Health Organization's standards;^16^ (2) performance status test (PST) score of 0–1; (3) no radiation or transarterial chemoembolization therapy used as anticancer therapy in the past; (4) macroscopic R0 resection, i.e., total macroscopic eradication of the tumor, the absence of identifiable intra‐ or extrahepatic metastatic lesions, and negative resection margins; (5) CTC sample collection and analysis 1 or 2 days before resection. Patients who had other cancers, died of perioperative factors, underwent non‐radical resection, and lacked preoperative CTC data or other data were excluded. According to the Clavien–Dindo classification, the severity of postoperative complications was determined, and the types of complications included post‐hepatectomy liver failure, abdominal bleeding, biliary fistulas, massive hydrothorax (>500 mL), intra‐abdominal infection, and severe wound infection. Patients who died during the perioperative phase, as a result of significant complications (Clavien–Dindo grade V), were excluded.

**FIGURE 1:**
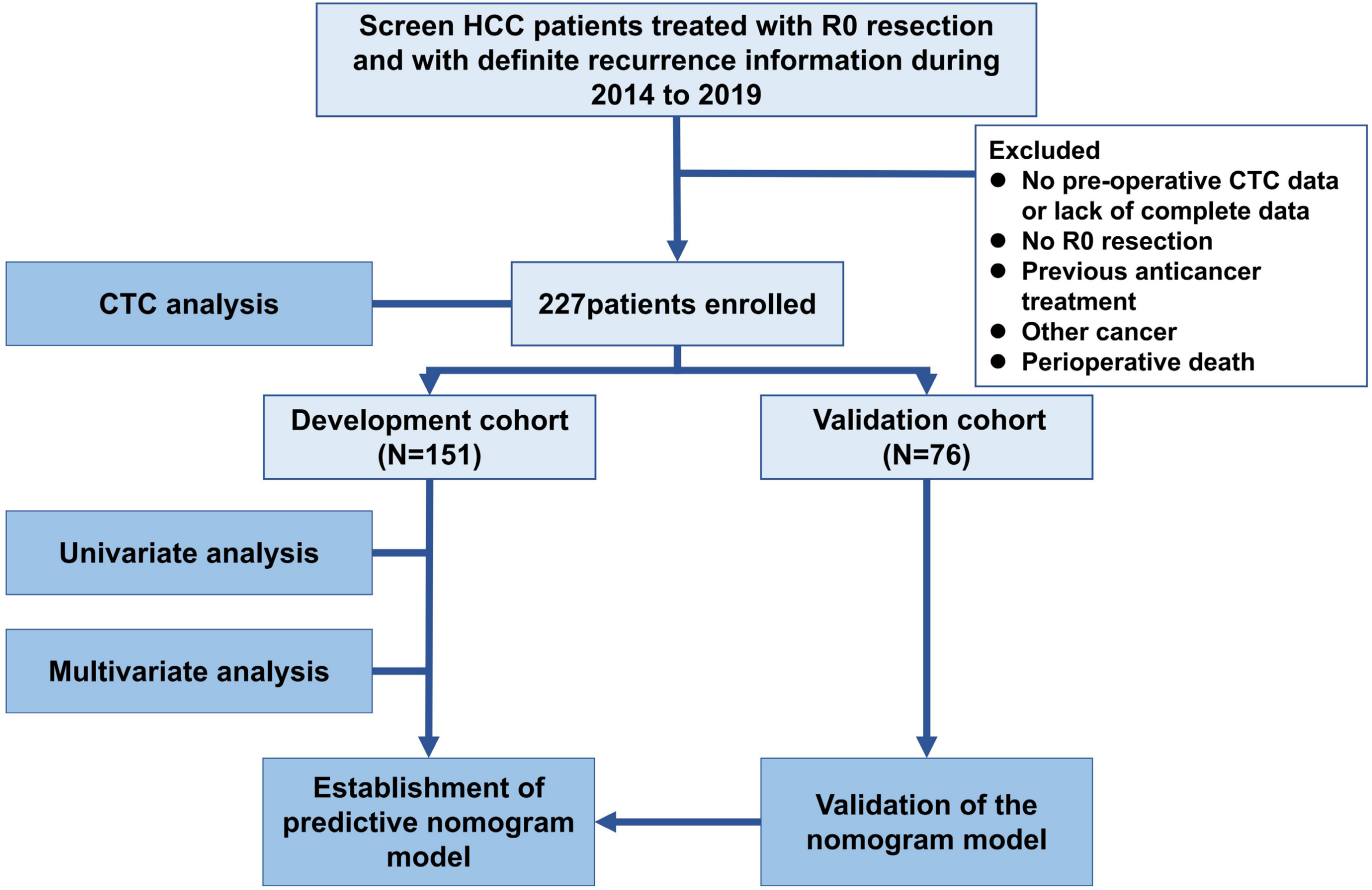
Flow chart of patient enrollment.

All patients (*n* = 227) were randomly divided into the development (*n* = 151) and validation (*n* = 76) cohorts at a ratio of 2:1. Cohort information and study design are shown in Figure 1.

The Guangxi Medical University Cancer Hospital's ethical committee approved the study protocol (No. LW2022154). Each patient provided their informed permission in writing.

### 2.2 Follow‐up assessment

For the first year after treatment, all patients were followed up on phone calls or outpatient services every 1–2 months, and then every 3 months thereafter. The following criteria confirmed recurrence: (1) Significantly elevated AFP levels in conjunction with one standard imaging modality (contrast‐enhanced ultrasonography, CT, or MRI); (2) if the AFP level is normal, at least two common imaging modalities simultaneously show the new lesion; (3) a new tumor biopsy that reveals a recurrent pathological diagnosis. Recurrence sites were classified as intrahepatic (including recurrence at the margin, adjacent segment, and distant segment) or extrahepatic(i.e., lung metastasis and bone metastasis). The endpoint of the follow‐up was June 30, 2021, with a median follow‐up of 28 months (range 3–84).

### 2.3 Isolation of CTCs by the CanPatrol system

The CanPatrol™ CTC‐enrichment technique was used to isolate the CTCs. Prior to hepatic resection, blood samples were collected 1 or 2 days beforehand. To eliminate skin cell contamination, blood samples from the peripheral circulation (5 mL ethylenediaminetetraacetic acid used to prevent blood clotting) were taken after the first 2 mL of blood was discarded.^7^, ^17^, ^18^ After removing the erythrocytes with red blood cell lysis buffer, the cells were resuspended in phosphate‐buffered saline with 4% formaldehyde for 5 min. The blood was then filtered using a system that includes a vacuum pump, an E‐Z96 vacuum manifold, a manifold vacuum plate with valve settings, and a filtration tube with an 8μm pore membrane. Filtration with a pressure of 0.08 Mpa pressure.^18^ mRNAs encoding EpCAM, CK8/18/19, Vimentin and Twist, were detected using in situ hybridization. The test was carried out on a 24‐well plate, and before hybridizing with the probe, the cells were given a protease treatment.

### 2.4 Statistical analysis

Statistical analysis of data was performed using Windows (IBM) versions of SPSS 26.0 and R (version 4.0.2, R Development Core Team, including the “survival” and “rms” packages).

The *t*‐test between‐group comparisons and chi‐squared (χ^2^) tests were used to evaluate the clinicopathological characteristics between the development and validation cohorts. Recurrence‐free survival (RFS) and overall survival (OS) were estimated using the Kaplan–Meier method and compared between groups using the log‐rank test. Based on variables in the development cohort, univariate and multivariate logistic regression analyses were performed to find independent predictors. In the univariate logistic regression analyses, potential predictors (*p* < 0.05) were further included in the subsequent multivariate regression analysis (using the forward likelihood ratio method, *p* < 0.05, as the entry and removal criteria). Nomogram models were established based on the results of the multivariable logistic regression analysis by the “rms” package in R software. The clinical utility of the nomogram was determined by decision curve analysis (DCA) after calculating the net benefits at each risk threshold probability. The clinical impact curve of the nomograms in the two cohorts was plotted to demonstrate the significance of each nomogram. All statistical assessments were two‐tailed, and *p* < 0.05 was considered statistically significant.

## 3 RESULTS

### 3.1 Survival analysis in patients with EHR versus without EHR

**FIGURE 2:**
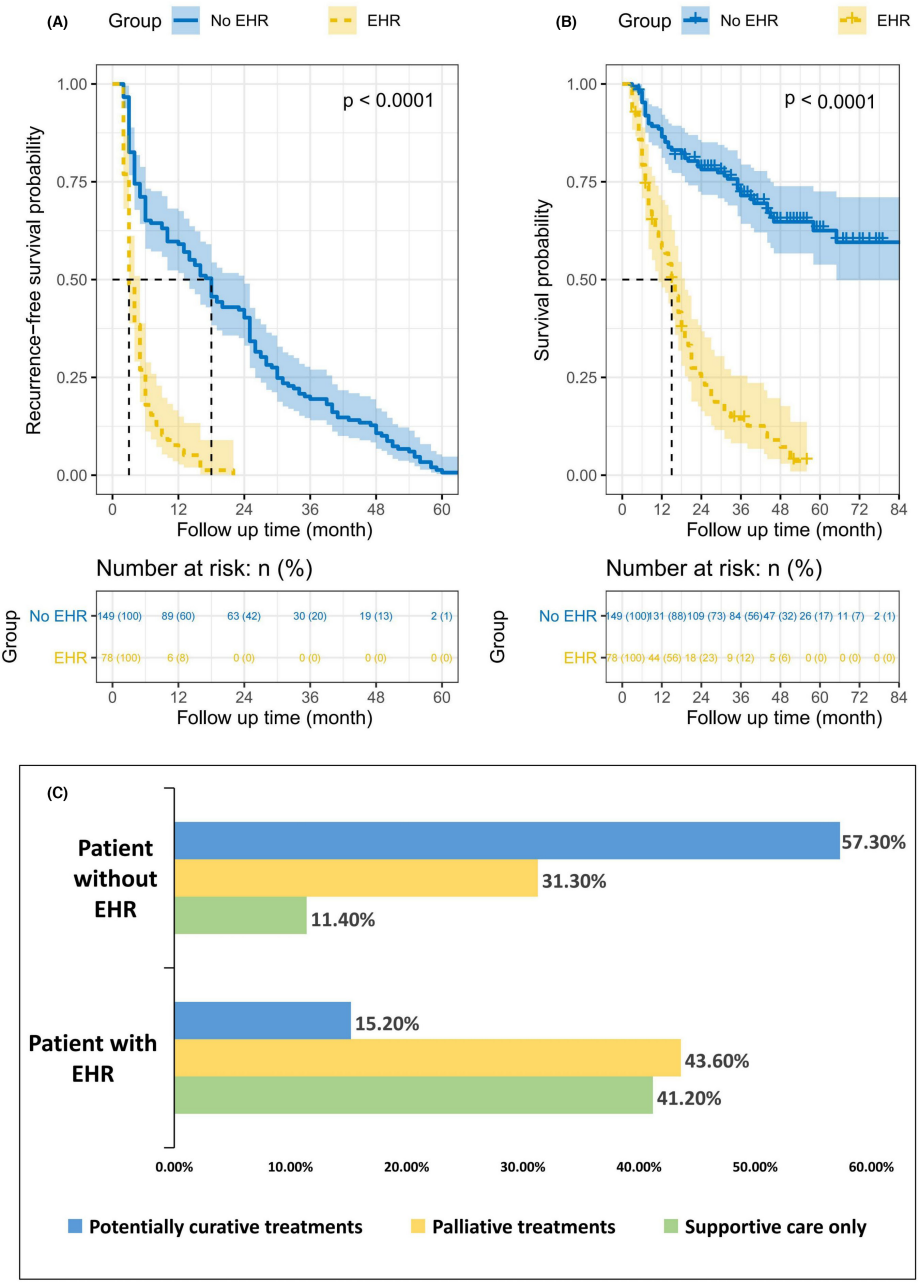
Survival analysis and post‐recurrence treatments in patients with extrahepatic recurrence (EHR) versus without EHR (*n* = 227). (A, B) Recurrence‐free survival and overall survival in patients with and without EHR. (C) Patients with and without EHR also differ in the subsequent modality of treatment.

Of the 227 patients, 78 had EHR (52 patients exhibited intrahepatic + EHR and 26 exhibited EHR alone). The log‐rank test showed that patients with EHR had significantly shorter RFS (median: 3 months vs. 18 months, HR: 3.639, *p* < 0.001) and OS (median: 15 months vs. 43 months, HR: 6.239, *p* < 0.001) than patients without EHR (Figure 2A,B). In addition, patients with and without EHR also differed in subsequent treatment modalities. More than half of the patients without EHR (57.3%) underwent potentially curative treatment, involving re‐hepatectomy, liver transplantation, local ablation, or a combination of potentially curative procedures. In contrast, the majority of patients with EHR (84.8%) only received palliative treatment or supportive care only (Figure 2C).

### 3.2 CTC count and type analysis between the patient with EHR and without EHR

**FIGURE 3:**
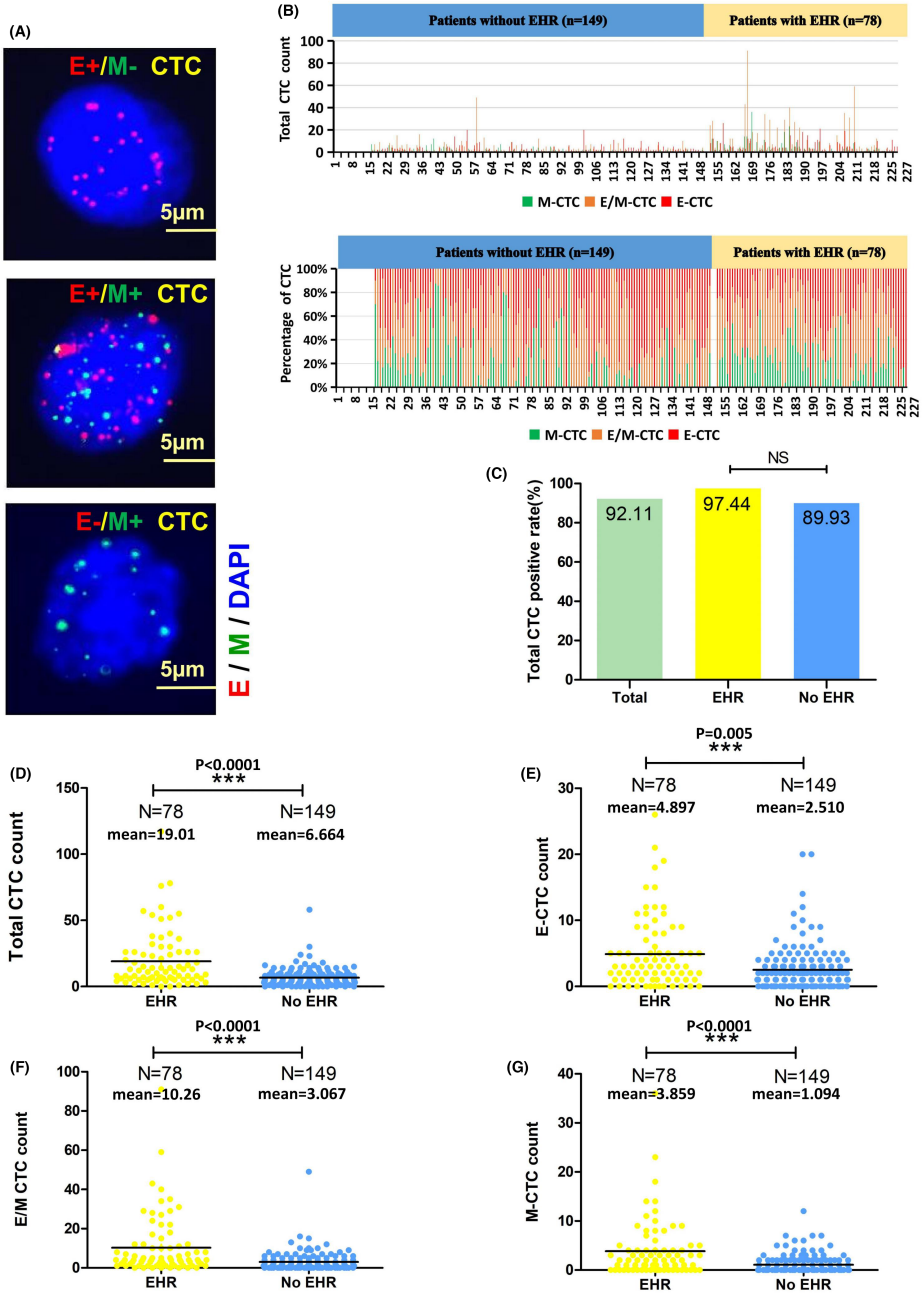
Status of circulating tumor cells (CTCs) undergoing epithelial‐mesenchymal transition before surgical resection in patients with and without EHR. (A) CTC subpopulations and their relationship with survival. CTCs were stained for epithelial markers (EpCAM and CK8/18/19, red fluorescence) and mesenchymal markers (Vimentin and Twist, green fluorescence) and classified as epithelial CTCs (E‐CTCs), epithelial/mesenchymal hybrid CTCs (E/M‐CTCs), and mesenchymal CTCs (M‐CTCs). (B)The total CTC count and the percentage of the CTC in the 227 patients. (C) The total CTC positive rate of the 227patiets and the subgroups of patients with and without EHR. Comparison of total CTC counts (D), E‐CTC counts (E), E/M CTC counts (F), and M‐CTC counts (G) in patients with and without EHR.

Using the CanPatrol CTC‐enrichment method, CTCs were found in blood samples from 227 patients. Gene expression in mesenchymal and epithelial cells is indicated by red and green fluorescent signals, respectively. A leukocyte marker called CD45 is expressed and is shown by a white fluorescence signal. Thus, three subpopulations were identified: (1) epithelial circulating tumor cell (E‐CTC), (2) epithelial/mesenchymal hybrid circulating tumor cell (E/M‐CTC), and (3) mesenchymal circulating tumor cell (M‐CTC) (Figure 3A). The CTC‐positive percentage was 92.11% (210/227), with 97.44% and 89.93% in patients with and without EHR, respectively (Figure 3B,C). The total CTC‐positive percentage did not significantly differ between the two subpopulations. However, the total CTC, E‐CTC, E/M‐CTC, and M‐CTC counts between patients with and without EHR were significantly different (all *p* < 0.001, Figure 3D–G). Furthermore, ROC curve analysis showed area under the curve (AUC) values of 0.728 (95% CI: 0.657–0.800; *p* < 0.001) for patient's total CTC count and the best cutoff value was 7 according to maximization of the Youden index.

### 3.3 Univariate and multivariate analyses to determine the factors for patients with EHR

Afterward, we explored the risk factors associated with EHR. First, all 227 patients were randomly divided into development (*n* = 151) and validation (*n* = 76) cohorts at a ratio of 2:1. Patient characteristics were similar in both cohorts (Table 1).

**TABLE 1:** A comparison of the characteristics of patients between the derivation set and validation set.

Variable	Development cohort (*N* = 151)	Validation cohort (*N* = 76)	*p*‐Value
Age (years)			0.884
≤45	68(45.0)	35(45.1)	
>45	83(55.0)	42(53.9)	
Gender			0.799
Female	20(13.2)	11(14.5)	
Male	131(86.8)	65(85.5)	
Total CTC count			0.736
>7	64(42.4)	34(44.7)	
≤7	87(57.6)	42(55.3)	
HBsAg			0.617
Positive	136(90.1)	70(92.1)	
Negative	15(9.9)	6(7.9)	
HBV‐DNA			0.511
>100	107(70.9)	57(75.0)	
<100	44(29.1)	19(25.0)	
AFP (ng/mL)			0.808
>400	86(57.0)	42(55.3)	
<400	65(43.0)	34(44.7)	
Tumor size (cm)			0.600
≥10	55(36.4)	26(34.2)	
5–9	67(44.4)	31(40.8)	
<5	29(19.2)	19(25.0)	
Edmondson grade			0.072
Low	51(33.8)	35(46.10	
High	100(66.2)	41(53.9)	
Node number			0.386
>3	32(21.2)	20(26.3)	
1–3	119(78.8)	56(73.7)	
Tumor capsule			0.499
Incomplete	45(29.8)	26(34.2)	
Complete	106(70.2)	50(65.8)	
Resection margin (cm)			0.729
>1	41(27.2)	19(25.0)	
<1	110(72.8)	57(75.0)	
MVD			0.482
High	84(55.6)	46(60.5)	
Low	67(44.4)	30(39.5)	
MVI			0.284
Positive	106(70.2)	48(63.2	
Negative	45(29.8)	28(36.8)	
PVTT			0.637
Positive	36(23.8)	16(21.1)	
Negative	115(76.2)	60(78.9)	
Liver cirrhosis			0.396
High/medium	34(22.5)	21(27.6)	
Low	117(77.5)	55(72.4)	
Postoperative complications			0.355
Yes	19(12.6)	13(17.1)	
No	132(87.4)	63(82.9)	
KI67	0.4194 ± 0.24039	0.3707 ± 0.26022	0.162

*Note*: (1) Total CTC counts were divided into two sub‐groups based on the best cutoff, patients classified as having low total CTC count (≤7) and having high total CTC count (>7). (2) MVD, MVI, and liver cirrhosis were determined by postoperative pathology. (3) PVTT is defined by preoperative imaging modality (contrast‐enhanced ultrasonography, CT, or MRI).

Abbreviations: AFP, alpha‐fetoprotein; HBsAg, hepatitis B surface antigen; HBV‐DNA, hepatitis B virus DNA; MVD, microvessel density; MVI, microvascular invasion; PVTT, portal vein tumor thrombosis.

In the development cohort, univariate analysis revealed that total CTC counts, AFP, tumor size, Edmondson grade, node number, tumor capsule, KI67, and MVI/PVTT status were significantly associated with EHR after hepatectomy (all *p* < 0.05). Incorporating the above variables into the multivariate analysis revealed that total CTC counts (HR, 1.977; 95% CI, 1.021–3.826; *p* = 0.014), node number (HR, 5.496; 95% CI, 2.020–14.956; *p* = 0.045), tumor size (HR, 2.922; 95% CI, 1.334–6.401; *p* = 0.028), and MVI (HR, 3.901; 95% CI, 1.706–8.921; *p* = 0.035) were associated with a greater risk of EHR (Table 2).

**TABLE 2:** Clinicopathological factors associated with EHR of hepatocellular carcinoma in the training cohort.

		Univariate analysis			Multivariate analysis	
Clinicopathological factors	Hazard ratio	95% CI	*p*	Hazard ratio	95% CI	*p*
Age (years)						
≤45 vs. >45	1.738	0.896–3.370	0.102	‐	‐	‐
Gender						
Female vs. male	1.369	0.530–3.538	0.517	‐	‐	‐
Total CTC count						
>7 vs. ≤7	4.407	2.143–8.656	0.001	2.928	1.243–6.894	0.014
HBsAg						
Positive vs. negative	1.811	0.548–5.982	0.330	‐	‐	‐
HBV‐DNA						
>100 vs. <100	0.755	0.369–1.542	0.440	‐	‐	‐
AFP (ng/mL)						
>400 vs. <400	2.923	1.444–5.917	0.003	0.961	0.376–2.454	0.933
Tumor size (cm)						0.028
≥10 vs. <5	49.000	6.190–387.902	0.000	15.875	1.762–143.053	0.014
5–9 vs. <5	13.689	1.747–107.272	0.013	7.599	0.882–65.461	0.065
Node number						
>3 vs. 1–3	6.133	2.581–14.576	0.000	2.934	1.026–8.392	0.045
Tumor capsule						
Incomplete vs. complete	3.164	1.537–6.517	0.002	0.785	0.287–2.147	0.637
Resection margin (cm)						
>1 vs. <1	0.576	0.266–1.246	0.161	–	–	–
MVD						
High vs. low	0.550	0.283–1.068	0.077	–	–	–
MVI						
Positive vs. negative	10.644	3.561–31.818	0.000	4.248	1.105–16.329	0.035
PVTT						
Positive vs. negative	2.178	1.1019–4.656	0.045	0.729	0.264–2.014	0.542
Liver cirrhosis						
High/medium vs. low	0.990	0.452–2.173	0.981	–	–	–
Postoperative complications						
Yes vs. no	1.944	0.739–5.118	0.178	–	–	–
KI67	25.620	5.577–117.705	0.000	5.155	0.691–38.445	0.110

Abbreviations: AFP, alpha‐fetoprotein; HBsAg, hepatitis B surface antigen; HBV‐DNA, hepatitis B virus DNA; MVD, microvessel density; MVI, microvascular invasion; PVTT, portal vein tumor thrombosis.

### 3.4 Nomogram models for EHR prediction

**FIGURE 4:**
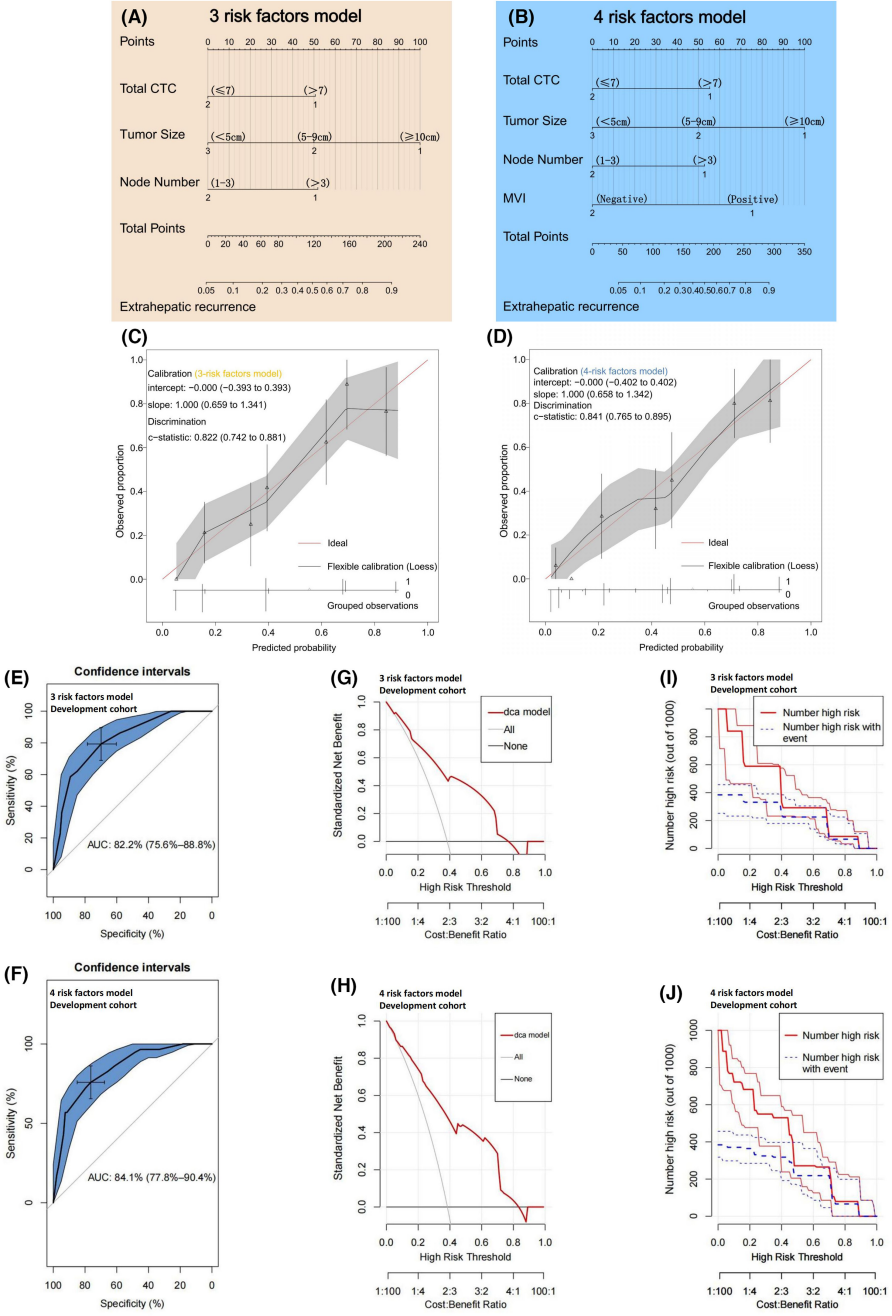
Extrahepatic recurrence (EHR) prediction nomogram models establishment and the predictive performance evaluation. (A, B) The nomograms of the three risk factors model and the four risk factors model. (C, D) Calibration curve for the three risk factors model and the four risk factors model in the development cohort. (E, F) ROC curves analyses indicated the good performance of EHR of the two nomogram models in the development cohort. Area under the curve (AUC) values of (E) 82.2% for the three risk factors model and (F) 84.1% for the four risk factors model in the development cohort. (G, H) DCA of the (G) three risk factors model and (H) four risk factors model for predicting EHR in the development cohort. (I, J) Clinical impact curves of the (I) three risk factors model and (J) four risk factors model for predicting EHR in the development cohort.

Among the four variables discussed above, three variables (total CTC count, tumor size, and node number) were available preoperatively. We established two nomogram models based on three risk factors and four total risk factors for preoperative and postoperative prediction, respectively (Figure 4A,B). The C‐index in the “three risk factors model” and “four risk factors model” were 0.822 and 0.841, respectively (Figure 4C,D). In the validation cohort, similar predictive performance was revealed. (Figure S1A,B). ROC curve analysis showed that the AUC values were 82.2% for the three risk factors model and 84.1% for the four risk factors model. The optimal cutoff score for pre‐operation was 100.3 (sensitivity: 67.8%; specificity: 80.5%) and 176.8 for post‐operation (sensitivity: 73.5%; specificity: 79.3%) (Figure 4E,F). A comparable predictive performance was observed in the validation cohort (Figure S1C,D). Using DCA, the two nomogram models can serve as valuable forecasting tools for predicting EHR in both cohorts (Figure 4G,H; Figure S1C,D). Clinical impact curves showed the nomogram's remarkable predictive potential (Figure 4I,J; Figure S1G,H).

### 3.5 Evaluation of the two nomogram models' therapeutic guidance capacity and survival analysis

**FIGURE 5:**
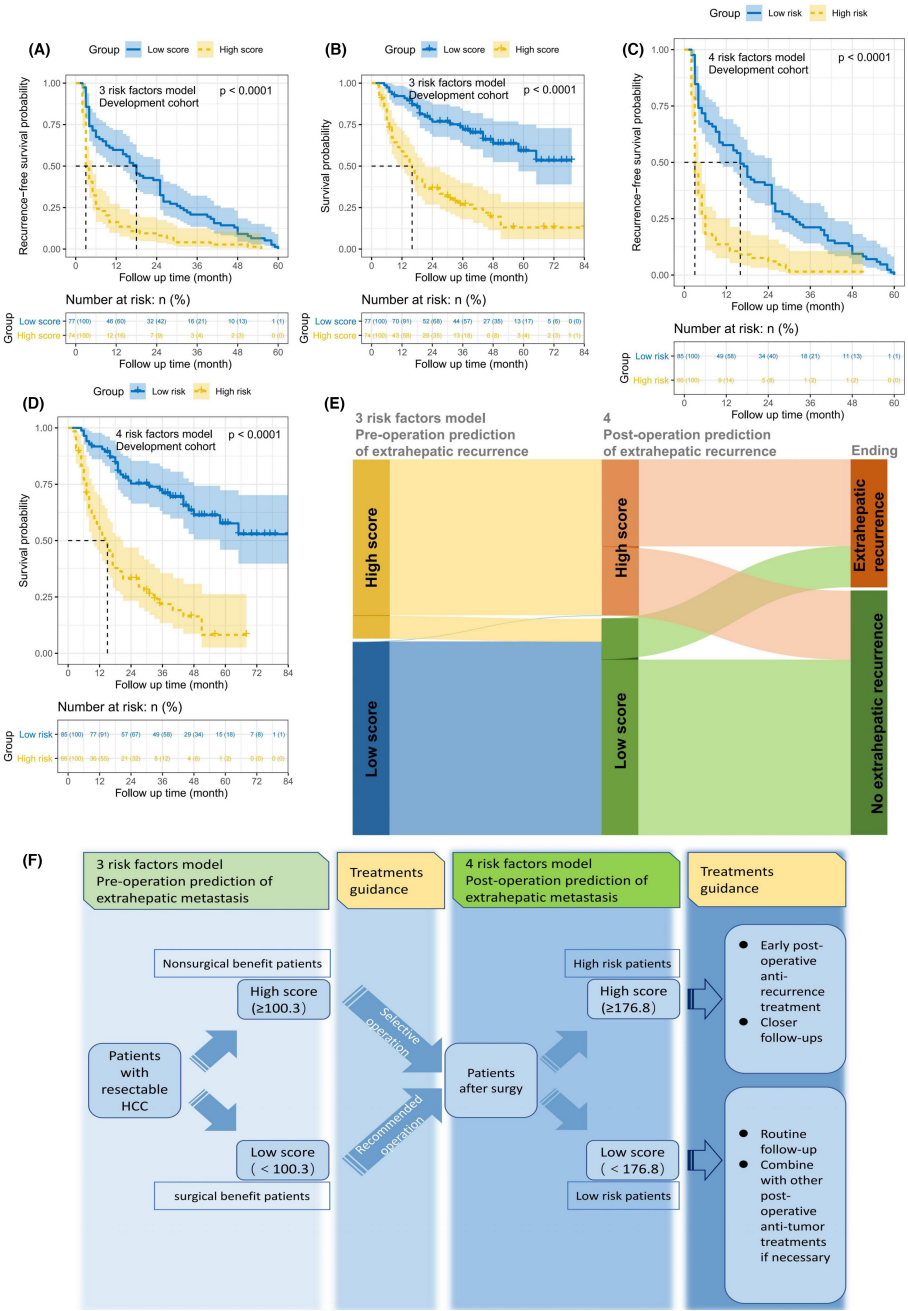
Evaluation of the two nomogram models' therapeutic guidance capacity and survival analysis. (A) Recurrence‐free survival (RFS) and (B) overall survival (OS) between the low score group and the high score group in the development cohort based on the three risk factors model. (C) RFS and (D) OS between the low score group and the high score group in the development cohort based on the four risk factors model. (E) Characteristics and outcome flow of 227 patients based on three risk factors model and four risk factors model. (F) Schematic diagram of potential therapeutic guidance value of the three risk factors model (presurgery) and four risk factors model (postsurgery).

With a threshold score of 100.3 before hepatectomy, patients with high scores had significantly shorter RFS and OS than those with low scores (Figure 5A,B). Likewise, with a score of 176.8 after hepatectomy, individuals with high scores had significantly worse RFS and OS than those with low scores (Figure 5C,D). Similar outcomes were attained in the validation cohort using the same cutoff score as in the validation cohort (Figure S1I–L).

As the Sankey diagram shows (Figure 5E), EHR occurred in 87.16% of patients assessed as high scoring in the three risk factors model, while the probability of EHR was 55.79% for patients with high scores in both models; in addition, the probability of no EHR for patients with low scores in the three risk factors model was 83.05% and 83.05% for patients with low scores in both models; In summary, both models demonstrated good predictive efficacy.

Consequently, the two nomogram models might be beneficial in directing HCC treatment methods (both preoperatively and postoperatively) (Figure 5F), but more evaluation in “real‐world” prospective multicenter research is still required.

## 4 DISCUSSION

To date, long‐term postoperative outcomes of HCC remain unsatisfactory owing to the multiplicity of recurrence patterns. EHR, although its incidence is lower than that of intrahepatic recurrence, often leads to a worse prognosis.^4^, ^9^ In the present study, we observed that HCC with EHR displayed an extremely low overall survival time, which may be due to not only the shorter time to recurrence but also the low curability percentage. Therefore, in order to achieve early prediction, it is clinically crucial to comprehend the associated risk factors for EHR.^8^, ^9^, ^11^, ^12^, ^13^, ^14^

Contrary to intrahepatic recurrence, which can either be a multicentric tumor developing de novo in the cirrhotic leftover liver or metastasis from the primary tumor, dissemination from the primary tumor preoperatively is the main reason for EHR.^4^, ^7^ Epithelial and mesenchymal cell‐specific biomarkers can be used to determine CTC count and morphologies, which is the foundation for CTC analysis, a crucial “liquid biopsy” technique being investigated for use in establishing treatment plans. In the present study, the positive rate of CTC detection did not differ significantly between the groups with and without EHR. This may explain the high incidence of CTC detection, regardless of EHR (97.44%) or without EHR (89.93%). In the pathogenesis of HCC, tumor spreading might be an initial event^4^, ^7^, ^19^; however, not all CTC‐positive patients have a poor prognosis, which also depends on the number and heterogeneity of CTCs.^20^ It is possible that some early disseminated CTCs may be low in number and “anoikis” quickly; and thus, do not result in EHR and poor prognosis. Theoretically, widespread preoperative metastasis is considered a prerequisite for EHR; Therefore, the likelihood of recurrence or metastasis increases with increasing CTC count. This was confirmed by this present study, which found that patients with EHR had significantly higher presurgical total CTC counts (>7) than those without EHR. Therefore, it is the CTC count instead of the positive rate that may reflect the risk of postoperative EHR. Nevertheless, CTCs may lose their epithelial properties and acquire mesenchymal traits during the epithelial‐mesenchymal transition (EMT), increasing their propensity for metastasis.^21^, ^22^ Therefore, positivity for M and E/M‐CTCs preoperatively may also increase the risk of extrahepatic metastasis, even in patients with low CTC counts.

In addition to preoperative CTC status, multivariate analyses in this study also revealed that large tumor size (>5 cm), multi‐nodule number (>3), and MVI status were risk factors in postoperative EHR. These findings are consistent with those of numerous studies,^10^, ^11^, ^12^, ^13^, ^14^ which, to a certain extent, suggest that for patients with EHR, the primary tumor has a high propensity for malignancy. However, most previous studies mainly focused on and were limited to the identification of risk factors. Furthermore, there is a limited degree of precision in using risk factors solely to predict EHR and create treatment plans due to the phenotypic and molecular heterogeneity of HCC. Therefore, an ideal postsurgical EHR predictive model should be generated based on the risk variables selected from these clinicopathological factors. To estimate each patient's likelihood of cancer recurrence or overall survival in HCC patients, a nomogram that incorporates various clinicopathological criteria is frequently utilized.^9^, ^10^ In this present study, based on the four variables, we established two nomogram models (preoperative and postoperative) to predict postsurgical EHR; both displayed good performance through multidimensional verification (e.g., calibration plot, ROC analysis, DCA, and clinical impact curve analysis). The preoperative prediction model used three available risk factors preoperatively (tumor size, node number, and preoperative CTC count), which may be used to formulate preliminary preoperative strategies (e.g., identify patients who are truly suitable for hepatectomy, who is so‐called “surgical benefit patients” and predict postsurgical survival). Given the possible limitations of preoperative predictive models, a postoperative prediction model incorporating all four variables was designed to guide postsurgical EHR prevention and treatment (e.g., postoperative monitoring, personalized treatment, and design of clinical trials). Moreover, the easy availability of four variables would aid the easy applicability of the two models in clinical practice.

This study had some limitations. First, all patients in the study came from a single center and the sample size was relatively small; therefore, there may be some bias in the statistical analysis. An external validation cohort in a multicenter study is needed in future studies to make the results more convincing. Second, this study was performed only at the clinical level, and the source of CTC counts was targeted only for patients with preoperative peripheral blood samples. Further studies on the molecular mechanisms are needed to elucidate the possible mechanisms underlying the influence of CTC notation on extrahepatic metastasis in patients with HCC.

## 5 CONCLUSIONS

Postsurgical EHR indicated very poor prognosis and was associated with four risk factors: high presurgical total CTC count, large tumor size (>5 cm), multi‐nodule number (>3), and MVI status. Subsequently, two nomogram models for postsurgical EHR prediction were established; these models showed relatively good predictive performance for EHR, which may benefit the clinical practice of HCC recurrence. Further multicenter studies are needed to assess the general applicability of the models.

## AUTHOR CONTRIBUTIONS

**Hao‐Wen Wei:** Conceptualization (equal); methodology (equal); writing – original draft (equal). **Shui‐Ling Qin:** Investigation (equal). **Jing‐Xuan Xu:** Data curation (equal); visualization (equal). **Yi‐Yue Huang:** Data curation (equal). **Yuan‐Yuan Chen:** Methodology (equal); project administration (equal); supervision (equal). **Liang Ma:** Funding acquisition (equal). **Lu‐Nan Qi:** Funding acquisition (equal); resources (equal).

## FUNDING INFORMATION

The National Nature Science Foundation of China (NSFC 81972306, 81502533, 82273405) and the Guangxi Nature Sciences Grants (2018GXNSFAA138028, 2018GXNSFAA050124) funded this research. This work was also funded in part by the Key Laboratory of Early Prevention and Treatment for Regional High Frequency Tumor, Ministry of Education/Guangxi, Independent Research Project (GKE202214).

## CONFLICT OF INTEREST STATEMENT

All authors have nothing to disclose.

## Supporting information


Figure S1.

## Data Availability

The author will unreservedly provide the original data supporting the conclusions of this article.
