# Supplementary material for: Nomograms for postsurgical extrahepatic recurrence prediction of hepatocellular carcinoma based on presurgical circulating tumor cell status and clinicopathological factors
Source: Cancer Med. 2023 Jun 20;12(14):15065–78. doi: 10.1002/cam4.6178 (PMC10417085; doi:10.1002/cam4.6178)
Supplement: Supplementary file 1 — Figure S1. [file CAM4-12-15065-s001.pdf]

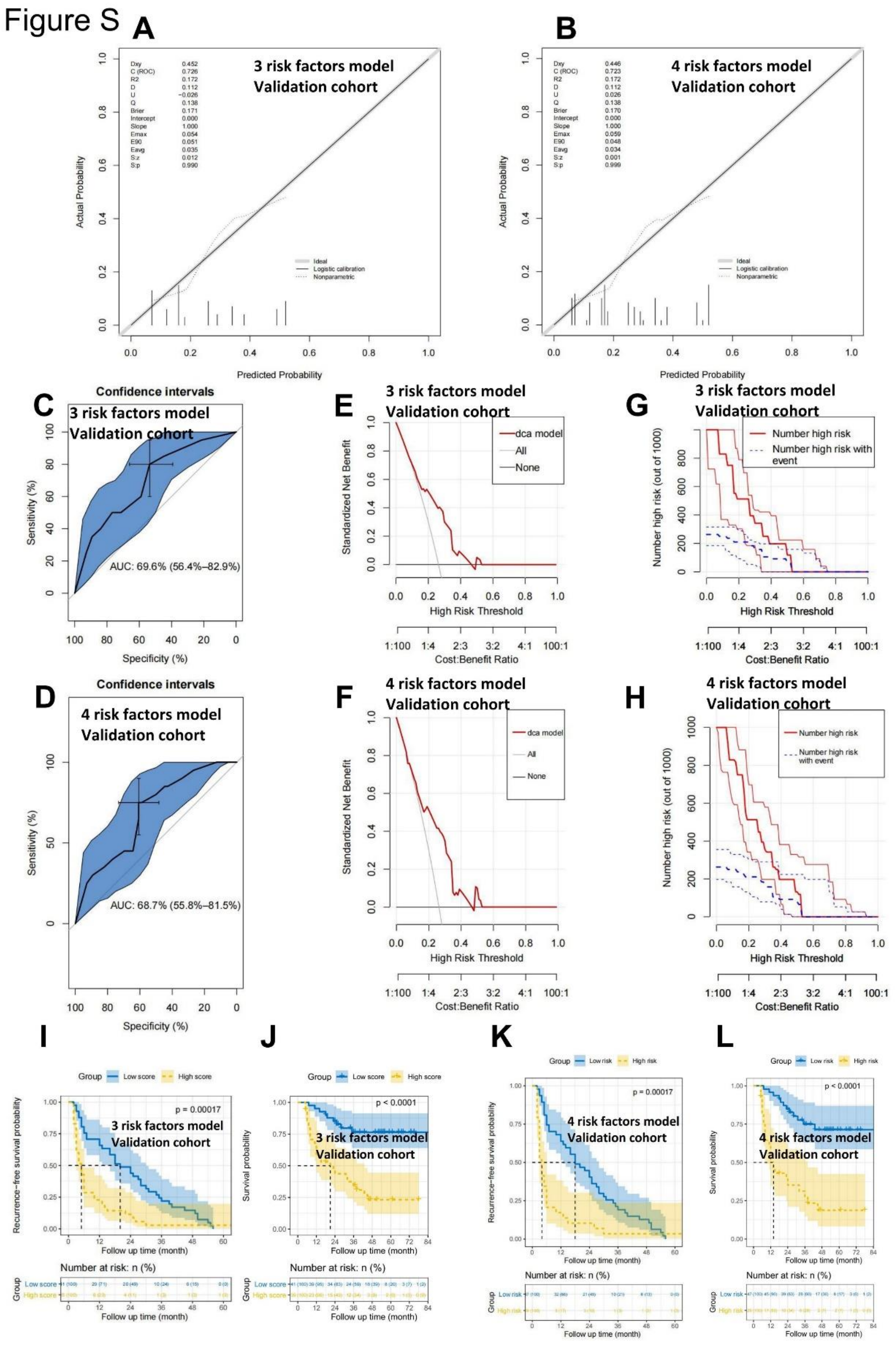

Figure S.

(A-B) Calibration plots for (A) “3 risk factors model” and (B) “8 risk factors model” in the validation cohort.

(C-D) ROC curves analyses indicated the good performance of EHR of the 2 nomogram models in the validation cohort. Under the curve (AUC) values of (C) 69.6% for the "3 risk factors model" and (D) 68.7% for the "4 risk factors model" in the validation cohort. (E-F) DCA of the (E) "3 risk factors model" and (F) "4 risk factors model" for predicting EHR in the validation cohort. (G-H) Clinical impact curves of the (G) "3 risk factors model" and (H) "4 risk factors model" for predicting EHR in the validation cohort. (I-J) The comparison of recurrence-free survival (RFS) and overall survival (OS) according to the optimal cut-off score of 100.3 pre-operation in the validation cohort. (K-L) The comparison of recurrence-free survival (RFS) and overall survival (OS) according to the optimal cut-off score of 176.8 post-operation in the validation cohort.
